# Food Rationing and National Efficiency

**Published:** 1918-06-15

**Authors:** 


### The Hospital

The Workers' Newspaper of Administrative Medicine and Institutional Life, Administration, National Insurance and Health.

No. 1671, Vol. LXIV. SATURDAY, JUNE 15, 1918. PRICE ONE PENNY

### FOOD RATIONING AND NATIONAL EFFICIENCY.

We have, in recent months, written so frequently on the subject of food that some apology might appear to be necessary for again returning to this topic. But our readers will allow that under the pressure of unprecedented circumstances food and its relation to individual and national welfare have taken on a new emphasis. In the days of peace its abundance was accepted as a commonplace by the enormous majority of people, and its arrival was unconsciously numbered among the events which require no foresight. Now all this is changed. In conversational circles food takes a leading position, and statesmen and politicians have brought it within the grasp of their administrative machinery. On a wide view, the fate of the world may be influenced by the success or failure of these methods, and in any event the individual citizen is conscious of the bond of the coupon. Patriotism is invoked for the restriction of appetite and the heavy weights become viewed with suspicion. What engages the interest of the tea-table, the club, and the market not unnaturally makes its appearance in the public press, and, as is the case with our colleagues, we are conscious of this pressure. We are conscious, also, of the responsibility of the journalist to endeavour to guide public opinion on right lines, and therefore we venture once more to survey the food problem as this has been modified by the practice of rationing.

At the outset we may say that we have no confidence in those who endeavour to regulate the ordinary habits of the nation by conclusions founded on laboratory experiments and chemical calculations. For the mere purpose of keeping the human machine running such conclusions are doubtless of value, but food subserves other functions than the physiological maintenance of tissues and organs at a certain level of activity. It has stimulating, creative, and æsthetic values, and the estimation of these defies the skill of the numerous and variegated crowd collected under the banner of the food expert. By instincts based upon experiences accumulated through countless generations men are guided to these wider values, and hence, at least to our judgment, the established food habits of a people have a presumptive wisdom and cannot safely be disturbed by doctrinal reasoning. What the physiologist has to do is not to prescribe our food habits but to explain them. As it is in this light we see the food question in general we can hardly be expected to sympathise with the writers who would fain persuade us that restricted supplies of food are an excellent discipline for the nation, and that under such discipline we are being led at last into the pathways of dietetic wisdom. If such teaching is intended to reconcile us to the necessities of the present position we can approve its motive. But we cannot accept it as scientific truth because, so it seems to us, it omits from its purview the large fact of human experience. Where man is free to choose, he will on the whole select what ministers to his welfare. Possibly in this choice there may be disadvantages, and if some scientific person can tell us how to avoid these without sacrificing the benefits associated with our selection we shall be much obliged to him. But to imply that man was incapable of the art of wise feeding prior to the arrival of the physiologist on the scene is to indict human intelligence and to place it on a lower level than that of our poor zoological relations.

Of course we have no desire to excite dissatisfaction with the existing rationing scheme. The responsible authority tells us it is a condition of national safety and we readily accept the consequences of the decision. Further, we agree that, keeping all the circumstances in view, the policy has been a wise one and that it has been administered with success. None the less, while accepting it as an unavoidable necessity, we do not for a moment agree with those who would have us believe that apart from the special claim of the day and hour a system of rationing is an excellent thing for the nation, for this would imply that a Government official is a better guide than the individual choice or instinct. No doubt it is probable, or even certain, that among individuals who habitually eat too much the existing order has been an influence for good. But that a parallel proposition relative to the workers of the nation is true we profoundly disbelieve. On the contrary, we are satisfied that harm is being done by restricted food supplies, and therefore, though we accept the necessity, we do not rejoice over it. On one point in particular we repeat our protest against the doctrine, said to be affirmed by the Government experts, that the brain-worker does not need more food than the idler. What calculations this conclusion is based on we do not pretend to know, but we have no hesitation in saying that it is contradicted by experience, and experience on such a point is worth more than a bushel of theories. Thus, to put the matter broadly, our position is that the system of rationing, though imposed on us by necessity and rightly enforced under a patriotic sanction, is nevertheless a national misfortune, and that in so far as it must be maintained it is a prejudice to the workers of the nation. The country will endure it to the extent and degree required by the cause for which the nation stands in this supreme struggle of the world's history. But do not let us be told that what is pressed upon us by necessity is really an excellent thing for our health and efficiency. Hard times we can accept with resolve, or even with cheerfulness, but cheap prattle about such experiences being "good for us" has a nauseating quality. The summons, it seems to us, is to encourage to endurance—so far as this may be necessary—and to teach the full utilisation of food by efficient and economical cookery. We have a great regard for the man of science, but at the moment the really helpful person on the food difficulty is the practical cook.

